# Effects of fixed and changing dietary forage neutral detergent fiber levels on ingestive behavior and blood biomarkers of Nellore bulls in the feedlot

**DOI:** 10.1093/tas/txag108

**Published:** 2026-07-20

**Authors:** Mailza Gonçalves de Souza, Filipe Araújo Canêdo Mendonça, João Pedro Azevedo Ventorin, Rafael Perches Guiducci, Matheus Sousa de Paula Carlis, Dante Pazzanese Duarte Lanna

**Affiliations:** Department of Animal Science, University of São Paulo - “Luiz de Queiroz” College of Agriculture, Piracicaba, SP 13418-900, Brazil; Phibro Animal Health Corporation, Teaneck, NJ 07666, United States; Department of Animal Science, University of São Paulo - “Luiz de Queiroz” College of Agriculture, Piracicaba, SP 13418-900, Brazil; Department of Animal Science, University of São Paulo - “Luiz de Queiroz” College of Agriculture, Piracicaba, SP 13418-900, Brazil; Department of Animal Science, School of Animal Science and Food Engineering, University of São Paulo, Pirassununga, SP 13635-900, Brazil; Department of Animal Science, University of São Paulo - “Luiz de Queiroz” College of Agriculture, Piracicaba, SP 13418-900, Brazil

**Keywords:** *Bos indicus*, feedlot cattle, rumination behavior, dietary fiber level, acute-phase proteins, dietary transition

## Abstract

This study investigated the effects of fixed and variable dietary forage neutral detergent fiber (fNDF) levels during the finishing feedlot period on ingestive behavior and blood biomarkers in Nellore bulls. Fifty-six Nellore bulls (initial BW: 460 ± 38 kg; age: 20 ± 1 months) were randomly allocated to four pens, with 14 animals per pen equipped with an automated feeding system (GrowSafe), the animal was considered the experimental unit. The study followed a completely randomized design. Bulls were fed for 100 d. dietary treatments were evaluated in two, fifty-day periods. In the first period (0–50 days), animals received diets with 12% or 7% fNDF. In the second period (51–100 days), one pen per treatment remained on the same diet, whereas the others switched fNDF levels (12%–7% step-down strategy or 7%–12% fNDF step-up strategy). Corn silage was used as the source of fNDF. Feeding, rumination, and resting behaviors were visually assessed by trained observers over 24 h on days 21, 42, 71, and 92. Blood samples were collected after each observation to determine lactate dehydrogenase (LDH), haptoglobin, and serum amyloid A (SAA) concentrations. Data were analyzed using the GLM procedure of SAS, considering a completely randomized design. Means were compared using Tukey’s test (*P* ≤ 0.05). In the first period, bulls fed 12% fNDF exhibited greater rumination at 21 (354.7 vs. 296.3 min/day; *P* = 0.02) and 42 days (363.6 vs. 254.2; *P* < 0.01). In the second period, on day 71, the increase from 7% to 12% fNDF step-up strategy raised rumination, while the decrease from 12% to 7% fNDF step-down strategy reduced rumination time (346.7 vs. 269 min/day; *P* = 0.02), with bulls maintained on continuous 12% or 7% fNDF exhibiting intermediate rumination times. On day 92, bulls maintained on 12% fNDF exhibited longer rumination times (343.3 vs. 285 min/day; *P* = 0.03). Idle time was greater in bulls fed 7% fNDF in period 1 (1037.4 vs. 935.2; *P* < 0.01). In period 2, switching 7%–12% fNDF step-up strategy reduced idle time (993.7), whereas 12%–7% fNDF step-down strategy increased it (1045; *P* < 0.01). On day 92, 12% fNDF exhibited lower idle time (935 vs. 1075; *P* < 0.01). Eating time was greater in bulls fed 12% fNDF at day 71 (*P* < 0.01). Chewing time was greater at 42 days (504.2 vs. 395.7; *P* < 0.01) and at 71 and 92 days for 12% (477.1 vs. 408; *P* < 0.01 and 493 vs. 351; *P* < 0.01). Regarding blood parameters, LDH concentration was lower for 12% fNDF at 71 (1808 vs. 2274 U/L; *P* < 0.01) and 92 days (*P* < 0.01). No differences were observed for haptoglobin or serum amyloid A (*P* > 0.05). During the first period (days 0–50), a significant interaction between rumination time and fNDF level was observed for LDH concentration (*P* < 0.01); in bulls fed 7% fNDF, LDH decreased linearly with increased rumination (*P* < 0.01). Haptoglobin was also affected (*P* < 0.01), decreasing with increased rumination in the 7% group (*P* = 0.02). Diets containing 12% fNDF promoted more favorable ingestive behavior and were associated with lower LDH concentrations, suggesting differences in physiological responses compared with 7% fNDF diets.

## Teaser

Dietary fiber level and fiber delivery strategy modulated behavioral and metabolic responses in feedlot finishing Nellore bulls. Feeding with 12% fNDF compared to 7% increased rumination and chewing activity while reducing inactivity and modifying selected blood biomarkers, potentially favoring ruminal adaptation during the feedlot.

## Introduction

To improve feed efficiency and carcass yield while reducing production costs, Brazilian feedlots have progressively increased concentrate inclusion, since forages are relatively more expensive when adjusted for energy content (Gentry et al. 2016). However, this greater reliance on starch-rich concentrates increases dietary energy density and the risk of ruminal acidosis, particularly in *Bos indicus* cattle, which have a smaller rumen, lower buffering capacity, and less adaptation to greater levels of soluble carbohydrates compared with taurine breeds (Phillips et al. 1960; Owens et al. 1998; Goulart 2008).

Despite this trend, forage inclusion remains essential for maintaining ruminal health (Galyean and Hubbert 2014). Adequate fNDF reduces the time ruminal pH remains below the subacute acidosis threshold and attenuates inflammation associated with endotoxin absorption (Plaizier et al. 2008; Khafipour et al. 2009). In addition, greater forage levels have been associated with improved dry matter intake (DMI), increased average daily gain (ADG), and reduced ruminal endotoxin production (Caetano et al. 2015; Marques et al. 2016).

Castillo-Lopez et al. (2014) demonstrated that the transition from forage-based diets (approx. 45.7% silage) to high-grain finishing diets (5% silage) significantly increases the risk and severity of ruminal acidosis. The study found that as steers progressed through the finishing period, the prevalence of acidosis (defined as pH < 5.5 for > 180 min/d) surged from 0.7% in the backgrounding phase to 37.8% in the final stage. This increased risk was strongly associated with the feeding phase and higher DMI, with each additional kg of DMI increasing the prevalence of acidosis by 6.88%. These findings highlight that prolonged exposure to finishing diets compromises ruminal health, which is negatively correlated with average ADG and feed efficiency, suggesting that strategies to regulate ruminal pH are essential for optimizing performance.

While roughage sources are essential to maintain rumen function and prevent digestive disorders, they are often not economically competitive compared to cereal grains in high-concentrate diets, making their inclusion justified primarily as a “functional” feed (Goulart et al. 2020). Consequently, feeding a fixed dietary fiber concentration throughout the entire finishing period might fail to balance animal health with economic efficiency. Dynamically changing the fNDF level during the feedlot phase emerges as a potential strategy to optimize this balance. For instance, a step-down transition (eg, starting with high fiber) may ensure initial ruminal adaptation and mat consistency before maximizing energy density to improve the economic outlook. Conversely, a step-up transition (increasing fiber late in the feeding period) could mitigate the cumulative digestive upset and metabolic stress that typically surge as days on feed increase.

However, these findings may not be directly applicable to *B. indicus* breeds, such as Nellore, because physiological and digestive differences between these subspecies may influence their responses to dietary fiber. These differences are believed to reflect evolutionary adaptations to environments with contrasting forage availability and quality. Consequently, although studies in taurine cattle have reported beneficial effects of both lower (Swanson et al. 2017) and higher dietary fiber concentrations have been reported to improve animal performance (Zinn et al. 2004; Caetano et al. 2015), specific recommendations for zebu cattle are still limited, reinforcing the need to establish dietary fiber requirements for animals finished in Brazilian feedlots.

In a previous study, Caetano et al. (2015) identified that approximately 12% fNDF represents the ideal level for feedlot cattle. However, national surveys (Silvestre and Millen 2021; Monsalve and Millen 2025) indicate many Brazilian feedlots only include 7% fNDF in their finishing rations. Thus, the two levels evaluated in the present study simultaneously represent current field practice and the level considered most suitable, based on scientific evidence.

Therefore, this study aimed to evaluate both the effects of dietary fNDF level and the effects of changing fNDF level during the feedlot period in Nellore bulls, with emphasis on ingestive behavior and inflammatory response. We hypothesized that bulls fed a constant low fNDF (7%) would exhibit impaired ingestive behavior and increased blood inflammatory markers compared with those who fed a constant high fNDF (12%). We further hypothesized that sequential transitions between these dietary fNDF levels would modulate these responses according to the final dietary fNDF concentration.

## Material and methods

All experimental procedures followed the guidelines established by the National Council for the Control of Animal Experimentation and were approved by the Ethics Committee on the Use of Animals of the Luiz de Queiroz College of Agriculture—USP (CEUA protocol no. 2833020223, ID 000421). The experiment was conducted at the Nutrigenomics Center of ESALQ, Piracicaba, São Paulo, Brazil.

### Animals and experimental design

Fifty-six Nellore bulls (IBW 460 ± 38 kg; age 20 ± 1 month; Table 1). At the start of the feedlot, the animals were subjected to a health protocol that included deworming (Ivomec MSD© São Paulo, Brazil), metaphylactic antibiotic treatment for respiratory diseases (Draxxin® [tulathromycin], Zoetis, Campinas, Brazil) and clostridial diseases (Poli Star© MSD, São Paulo, Brazil), and administration of a vitamin complex containing vitamins A, D, and E (Vigantol© Bayer, São Paulo, Brazil).

**Table 1:** Initial and final weight of the animals.

Item	Treatment				SEM^e^	*P*-value
	12% fNDF^a^	7% fNDF^b^	12%–7% fNDF step-down strategy^c^	7%–12% fNDF step-up strategy^d^		
**Live weight (kg)**						
** d 0 days**	464.5	462.7	460.0	454.0	5.41	0.91
** d 0–50 days**	547.4	536.7	544.4	532.4	4.56	0.83
** d 0–100 days**	630.1	613.2	625.5	617.6	4.97	0.62

In the first period (0–50 days), animals received diets containing 12% fNDF (two pens) or 7% fNDF (two pens), and results were averaged across pens with the same fNDF level.

a 12% fNDF—constant 12% fNDF throughout the 100 days.

b 7% fNDF—constant 7% fNDF throughout the 100 days.

c 12%–7% fNDF step-down strategy—step-down strategy fNDF, from 12% in period 1 to 7% in period 2.

d 7%–12% fNDF step-up strategy—step-up strategy fNDF, from 7% in period 1 to 12% in period 2.

e SEM, standard error of the mean.

**Figure 1:**
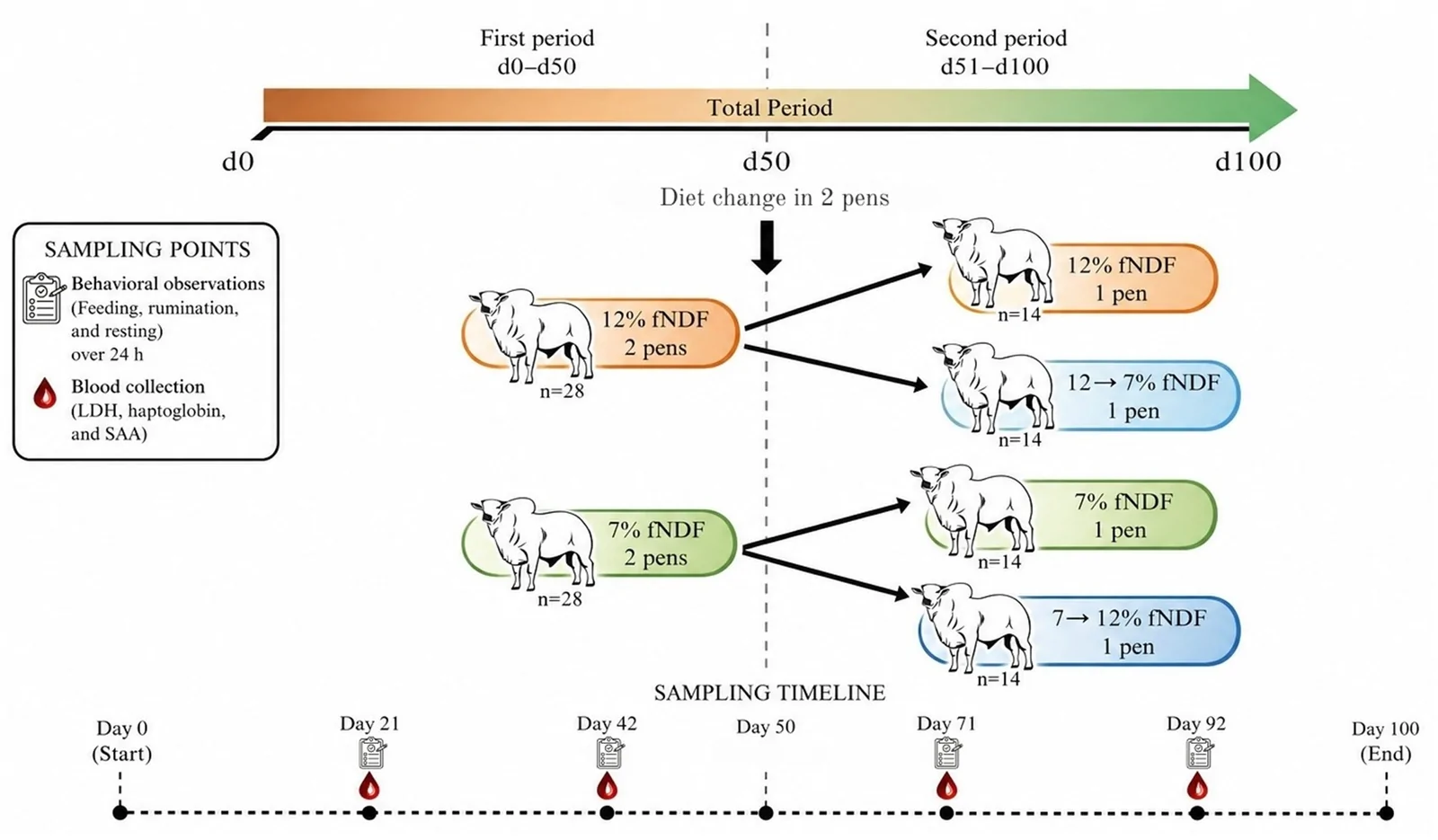
Experimental design and timeline of the feeding trial. The 100-day experiment was divided into three periods: the first (0–50 d), the second (51–100 d), and the overall (0–100 d). During the first period, animals were assigned to diets containing either 12% or 7% forage neutral detergent fiber (12% fNDF or 7% fNDF; DM basis), with 28 animals per treatment distributed across four pens, two per treatment. On day 50, two pens were redistributed to the diet, and two continued with the identical diets, resulting in four treatments in the second period (14 animals per treatment; one pen each): 12% fNDF, 7% fNDF, 12%–7% step-down, and 7%–12% step-up. This design allowed the evaluation of constant versus changing fNDF strategies and the effects of increasing or decreasing dietary fiber levels during the feedlot trial.

The experiment was conducted from May to September 2022, spanning 121 days. A common 21-day adaptation period was applied to all bulls during which they were adapted to the facilities and diets according to the adaptation scheme presented in Table 2. This adaptation phase was identical for all treatment groups and was not considered part of experimental treatments. After the adaptation period, the dietary treatments were imposed and evaluated for 100 days under feedlot conditions. During the first period (days 0–50), two dietary treatments were evaluated: high-concentrate diets containing either 12% or 7% fNDF on a DM basis. Four treatments were applied during the second period (days 51–100). Two pens maintained the original treatments from the first period (12% or 7% fNDF), while one pen that initially received 12% was switched to a reduced 7% fNDF level, and another pen that started with 7% was changed to an increased 12% fNDF level. A schematic representation of the experimental design is presented in Fig. 1.

**Table 2:** Nutritional composition of ingredients used in experimental diets (% DM).

Items	Corn silage	Ground corn	Cottonseed	Soybean meal
**Ingredients (% of dry matter)**				
** Dry matter**	42.2	89.2	89	89.2
** Crude protein**	10.0	9.2	26.6	47.7
** Ash**	6.6	2.7	4.0	8.6
** NDF^a^**	51.5	12.4	57.8	12.9
** ADF^b^**	33.0	3.6	46.2	6.4
** Starch**	20.4	66.5	—	—
** Ether extract**	4.1	6.3	14.9	2.6

a NDF, neutral detergent fiber.

b ADF, acid detergent fiber.

The experiment was conducted as a completely randomized design, considering the individual animal as the experimental unit. Each animal was fitted with an electronic identification tag compatible with the GrowSafe system (Lenexa, KS, USA). The GrowSafe system is a validated and widely used automated technology that continuously records individual feeding behavior and feed intake of animals housed in group pens through radio-frequency identification (RFID), thereby allowing reliable individual measurements under collective housing conditions. Each pen was equipped with two GrowSafe feeding bunks, with each bunk designed to accommodate up to eight animals, according to the manufacturer’s recommendations. Group housing was adopted to allow animals to express their natural social and feeding behaviors, closely resembling commercial production systems. Each of the four collective pens measured 10 × 16.75 m (167.5 m^2^), providing approximately 12.0 m^2^ per animal. Throughout the experiment, animals remained on their assigned dietary treatments and were randomly rotated among pens every 14 days to minimize potential confounding effects associated with pen location, including differences in solar radiation, airflow, shade, and other microenvironmental conditions. This management strategy was implemented to reduce environmental bias and increase the robustness and reliability of experimental data. Fourteen animals were housed in each pen throughout the experimental period.

### Feed composition and daily management

Feed analyses (Table 3) quantified starch, NDF, ADF, ash, fatty acids, and crude protein (CP). Crude protein was determined by combustion analysis using a Leco FP-200 (Leco Corp., St. Joseph, MI). Chemical analyses were performed according to the guidelines of the Association of Official Analytical Chemists (AOAC 2000), while the NDF and ADF contents were determined by Van Soest et al. (1991). Dry matter of the experimental diets was also determined using a forced-air oven at 105 °C (AOAC Method 934.01). Daily silage samples were analyzed for dry matter to adjust the daily diet.

**Table 3:** Composition of diets used during the adaptation period (% DM).^a^

Ingredients	1–7 days	8–14 days	15–21 days
**Corn silage**	96.7	48.0	28
**Ground corn**	–	34.8	54.7
**Cottonseed**	–	10.0	10.0
**Soybean meal**	2.3	3.0	3.0
**Urea**	0.4	1.3	1.3
**Mineral premix^b^**	0.5	2.7	2.7
**Potassium chloride**	–	0.20	0.25

a Dry matter.

b Mineral premix—was included in all experimental diets. Contained (per kg of product): Ca, 211–316 g; P, 13 g; Na, 61 g; Mg, 19 g; S, 24 g; Zn, 2,220 mg; Mn, 1,650 mg; Cu, 556 mg; Fe, 371 mg; I, 28 mg; Co, 11 mg; Se, 7.4 mg; vitamin A, 93,000 IU; vitamin D3, 15,000 IU; vitamin E, 138 mg; monensin, 928 mg; herbal flavoring, 5.4 mg; onion extract, 9.4 mg; and grape seed extract, 1.7 mg.

The 100-day experimental period used the diets presented in Table 4, with varying levels of fNDF inclusion. The diets were mixed using an American Calan® mixer wagon for 5 minutes (Northwood, New Hampshire, USA). The particle size distribution of the corn silage (Table 3) was determined using a sieve box (Santa Fé Agroinstituto, PR, Brazil) according to the method described by Lammers et al. (1996). Physically effective neutral detergent fiber (peNDF) was analyzed according to Mertens (1997). The diet was offered twice a day, 50% at 0700 h and 50% at 1600 h, and prepared just before feeding. Leftovers were weighed daily to adjust the amount provided. Diets were formulated using RLM software, version 3.399 (Lanna et al. 1999) to meet requirements for protein, rumen-degradable protein, minerals, and vitamins.

**Table 4:** Composition of experimental diets (% DM).

Ingredients	Diets^a^	
	12% fNDF^a^	7% fNDF^a^
**Corn silage**	23.3	13.6
**Ground corn**	64.0	73.5
**Cottonseed**	7.0	7.0
**Soybean meal**	1.5	1.5
**Urea**	1.2	1.2
**Mineral premix**	2.7	2.7
**Potassium chloride**	0.2	0.5
**Chemical composition**		
** Dry matter**	73.0	80.0
** Ether extract**	6.0	6.2
** Crude protein**	14.1	14.1
** Rumen degradable protein**	9.6	9.4
** Starch**	47.3	51.6
** Neutral detergent fiber**	24.1	20.3
** Acid detergent fiber**	13.3	10.4
**Particle size**		
** 19 mm**	1.06	0.62
** 8 mm**	31.2	21.7
** 4 mm**	5.2	4.2
** Particles less than 4 mm**	62.4	73.3
**peNDF^b^**	7.79	4.54

a fNDF—Neutral detergent fiber from forage; 12% fNDF—constant 12% fNDF throughout the 100 d. 7% fNDF—constant 7% fNDF throughout the 100 d.

b peNDF, The physically effective NDF (peNDF) of the total diet was calculated as the NDF concentration in the diet multiplied by the proportion of particles retained on sieves ≥ 8 mm using the Penn State Particle Separator (Mertens 1997).

### Ingestive behavior

Animals were observed for 24 consecutive hours (1700–1700 h) on days 21, 42, 71, and 92. Observers responsible for ingestive behavior assessment were previously trained according to standardized criteria, and all had prior experience in assessing bovine behavior. During this period, Idleness was defined as the period during which animals were neither feeding, ruminating, nor drinking. Feeding, rumination, and idle time (min/day) were recorded. Behavioral states were recorded using instantaneous scan sampling at 10-min intervals as described by Goulart et al. (2020).

Total chewing time was calculated as the sum of eating time and rumination time (Maekawa et al. 2002; Kononoff et al. 2003). Based on the recorded values, the following parameters were calculated for each animal: daily feed intake time (min/day), daily rumination time (min/day), daily idle time (min/day), and daily chewing time (min/day) (Maekawa et al. 2002).

### Blood parameters

Blood samples were collected on days 21, 42, 71, and 92 for lactate dehydrogenase (LDH) analysis, and on days 21 and 92 for serum amyloid-A and haptoglobin analysis. Samples were obtained via jugular venipuncture using 10 mL Vacutainer® tubes, centrifuged at 3000 rpm for 10 minutes at 4 °C, and the serum was stored in Eppendorf tubes at −20 °C, as described by Kerr (2002), for later analysis. LDH was measured using an automated biochemistry system (Model SBA–200, CELM, Barueri–SP, Brazil) with LABTEST Diagnóstica SA kits (Lagoa Santa–MG, Brazil). Haptoglobin concentrations were determined using an ELISA kit following the methods of Makimura and Suzuki (1982) and Cooke and Arthington (2013). Serum amyloid A was analyzed using a commercial ELISA kit (Bovine Cattle SAA, catalog ELK5620, ELK Biotechnology, Doylestown, PA).

### Statistical analysis

All statistical analyses were performed using SAS University Edition (SAS Studio, SAS OnDemand for Academics). The data were first assessed for normality of residuals and potential outliers using the UNIVARIATE procedure, which generated and evaluated studentized residuals.

The behavioral and blood parameter data were analyzed using the General Linear Model procedure (PROC GLM) of SAS. Because the animals were rotated systematically among pens to homogenize environmental variance, and individualized data were collected using the GrowSafe system, the study was analyzed as a completely randomized design considering the animal as the experimental unit. The statistical model included dietary treatment as the sole fixed effect.

Results are presented as east squares means (LSMeans) and standard errors of the mean (SEM), obtained through the PROC MEANS procedure. To evaluate the differences among treatments, means were compared using the Tukey’s test. Treatment differences were considered statistically significant at *P* ≤ 0.05. Statistical tendencies were not considered in this evaluation.

The regression analyses were performed to investigate the relationships between rumination time and serum concentrations of lactate dehydrogenase (LDH) and haptoglobin. For each response variable, a linear model was fitted including the fixed effects of dietary fNDF level (7 or 12%), rumination time (continuous covariate), and the interaction between dietary fNDF level and rumination time. When the interaction was significant, treatment-specific regression coefficients were estimated, and the fitted equations are presented in the “Results” section.

## Results

**Figure 2:**
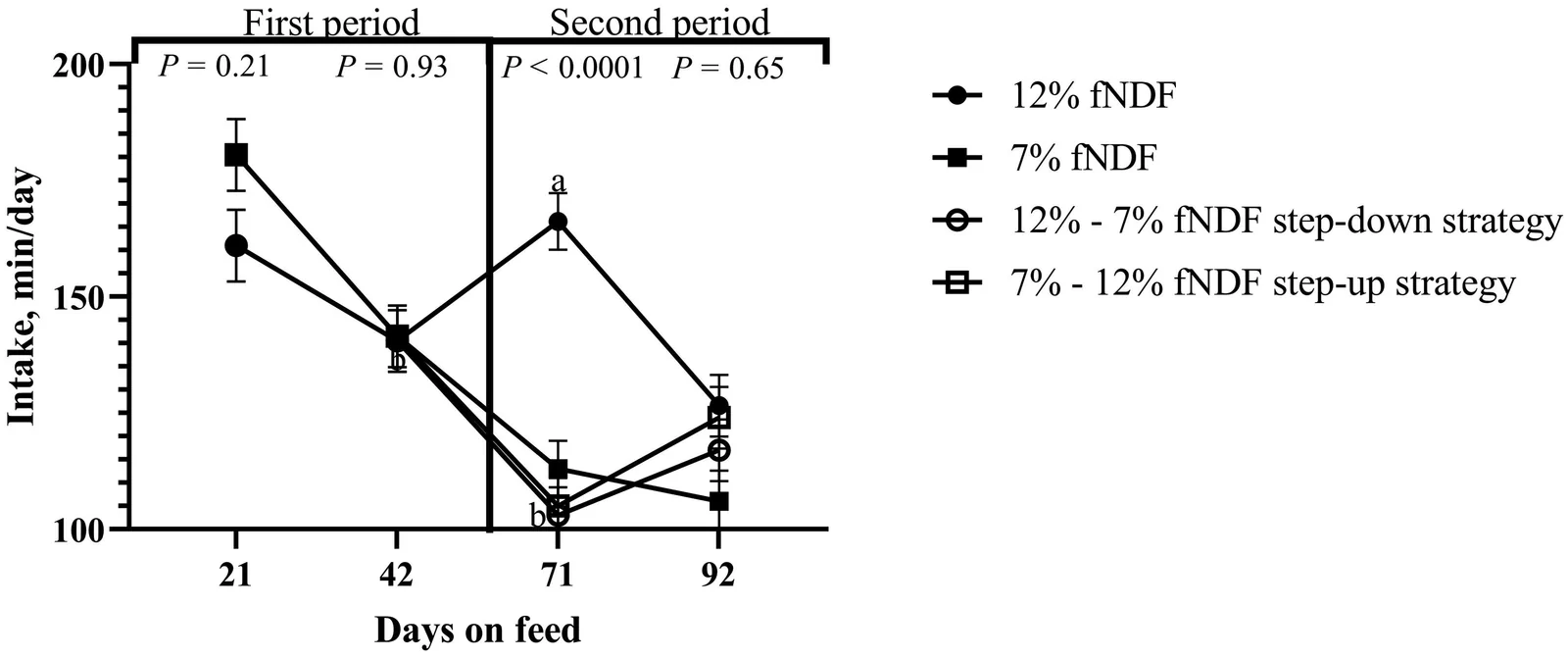
Intake time (min/day) of Nellore bulls fed diets containing 12% or 7% forage neutral detergent fiber (fNDF), either continuously or with dietary transitions (12%–7% fNDF step-down strategy or 7%–12% fNDF step-up strategy), measured at 21, 42, 71, and 92 days of feedlot. 12% fNDF—constant 12% fNDF throughout the 100 days. 7% fNDF—constant 7% fNDF throughout the 100 days. 12%–7% fNDF step-down strategy—step-down strategy fNDF, from 12% in period 1 to 7% in period 2; 7%–12% fNDF step-up strategy—step-up strategy fNDF, from 7% in period 1 to 12% in period 2. In the first period (0–50 days), animals received diets containing 12% fNDF (two pens) or 7% fNDF (two pens), and results were averaged across pens with the same fNDF level. ^a,b^Means within a row with different superscript letters differ (*P* < 0.05) according to Tukey’s test.

All performance outcomes evaluated in this study are reported in Souza et al. (2026). Feeding time (min/d) did not differ (*P* > 0.05) among treatments during the first period or at 92 d of the feedlot (Fig. 2). However, at 71 d, bulls fed 12% fNDF exhibited longer feeding times than other treatments (*P* < 0.01).

**Figure 3:**
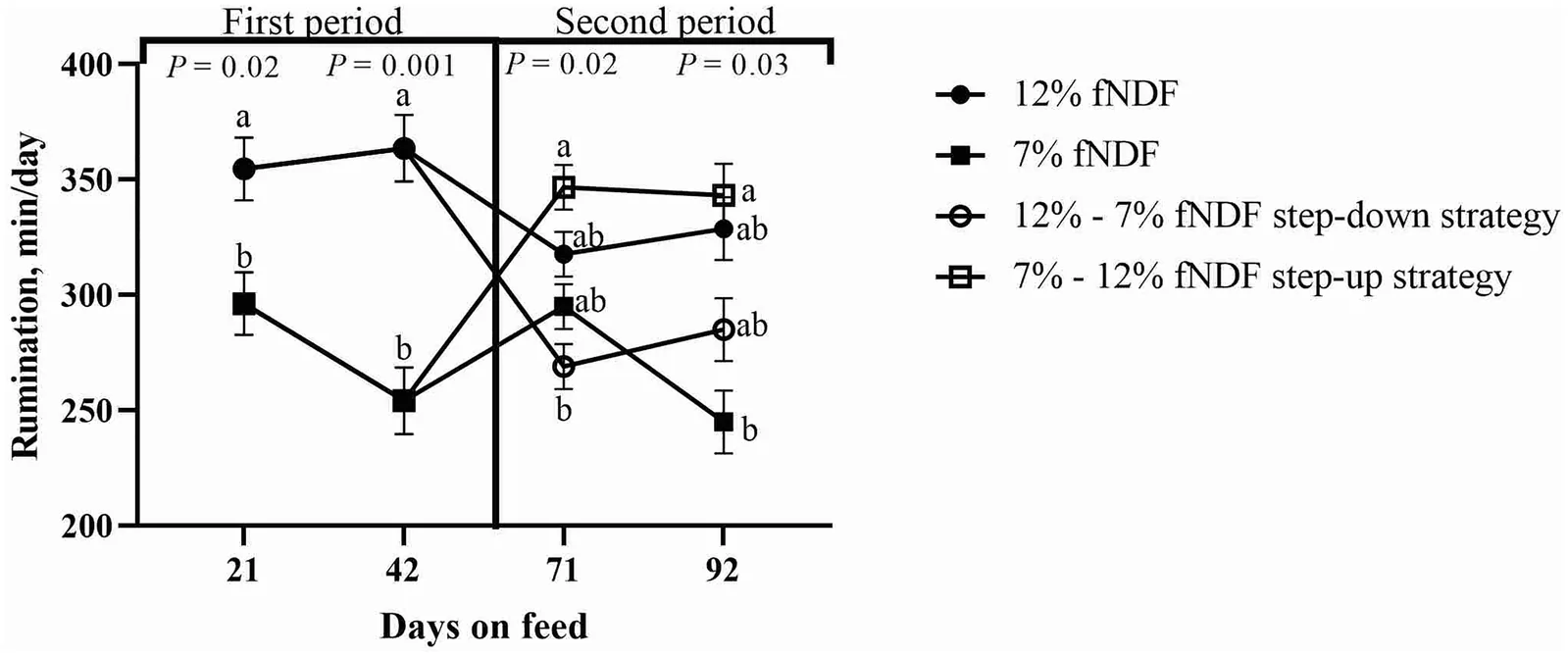
Rumination time (min/day) of nellore bulls fed diets containing 12% or 7% forage neutral detergent fiber (fNDF), either continuously or with dietary transitions (12%–7% fNDF step-down strategy or 7%–12% fNDF step-up strategy), measured at 21, 42, 71, and 92 days of feedlot. 12% fNDF—constant 12% fNDF throughout the 100 days. 7% fNDF—constant 7% fNDF throughout the 100 days. 12%–7% fNDF step-down strategy—step-down strategy fNDF, from 12% in period 1 to 7% in period 2; 7%–12% fNDF step-up strategy—step-up strategy fNDF, from 7% in period 1 to 12% in period 2. In the first period (0–50 days), animals received diets containing 12% fNDF (two pens) or 7% fNDF (two pens), and results were averaged across pens with the same fNDF level. ^a,b^Means within a row with different superscript letters differ (*P* < 0.05) according to Tukey’s test.

During the first period, bulls fed 12% fNDF exhibited longer daily rumination times (min/d) compared to those fed 7% fNDF (*P* < 0.05; Fig. 3). In the second period, after the dietary transition, bulls assigned to the 12%–7% Step-down strategy exhibited a reduction in rumination time (*P* = 0.02) compared to those on the 7%–12% Step-up strategy, which displayed the longest rumination times at 71 and 92 d. Bulls maintained on continuous diets (12% or 7% fNDF) exhibited intermediate rumination times at 71 d. At 92 d, this pattern persisted, with bulls fed the continuous 7% fNDF exhibiting the shortest rumination time compared to the 7%–12% Step-up group.

**Figure 4:**
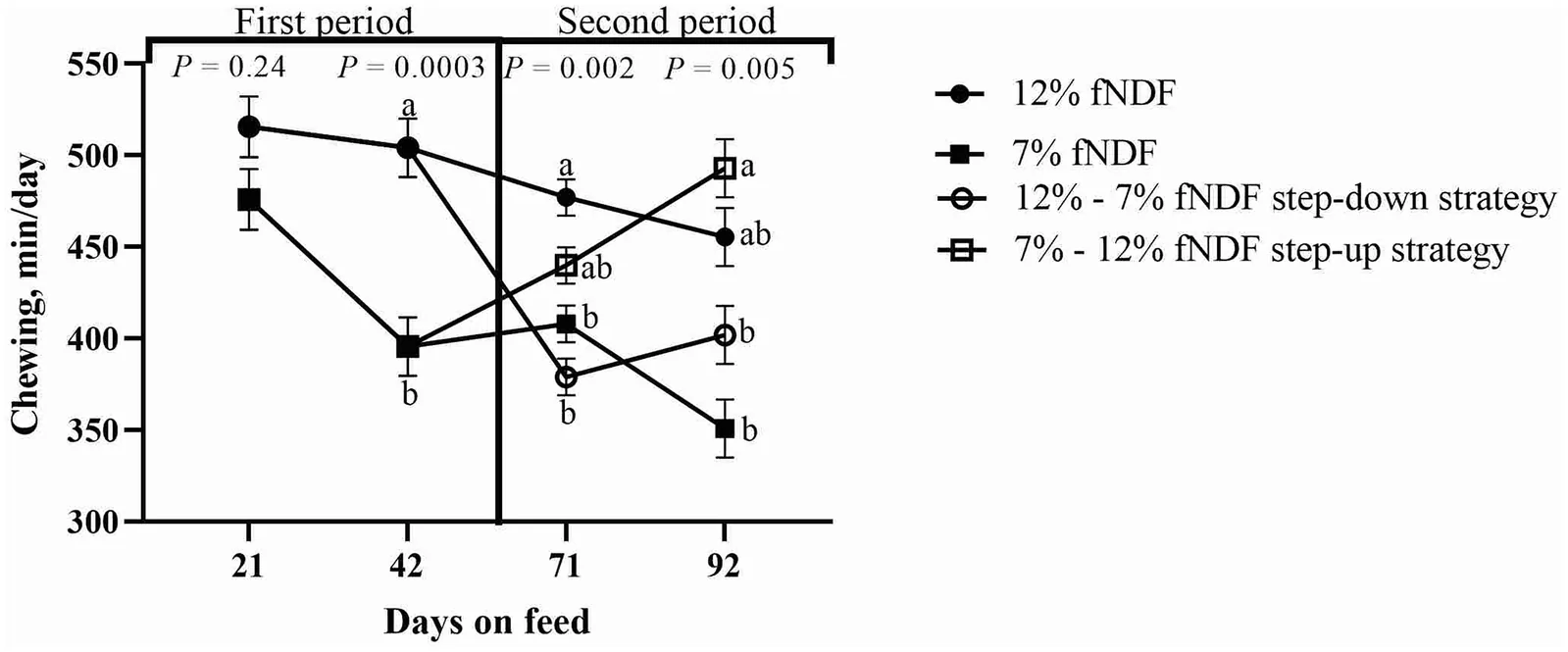
Chewing time in minutes per day of Nellore bulls fed diets containing 12% or 7% forage neutral detergent fiber (fNDF), either continuously or with dietary transitions (12%–7% fNDF step-down strategy or 7%–12% fNDF step-up strategy), measured at 21, 42, 71, and 92 days of feedlot. 12% fNDF—constant 12% fNDF throughout the 100 days. 7% fNDF—constant 7% fNDF throughout the 100 days. 12%–7% fNDF step-down strategy—step-down strategy fNDF, from 12% in period 1 to 7% in period 2; 7%–12% fNDF step-up strategy—step-up strategy fNDF, from 7% in period 1 to 12% in period 2. In the first period (0–50 days), animals received diets containing 12% fNDF (two pens) or 7% fNDF (two pens), and results were averaged across pens with the same fNDF level. ^a,b^Means within a row with different superscript letters differ (*P* < 0.05) according to Tukey’s test.

Total chewing time (min/d) did not differ (*P* = 0.24) during the first 21 d (Fig. 4). However, bulls fed 12% fNDF exhibited longer chewing times than those receiving 7% fNDF at 42 d (*P* < 0.01). At 71 d, bulls fed 12% fNDF maintained a longer chewing time (*P* < 0.01) compared to the 7%–12% Step-up group. At 92 d, bulls on the 7%–12% Step-up strategy maintained longer chewing time than those fed the continuous 7% fNDF.

**Figure 5:**
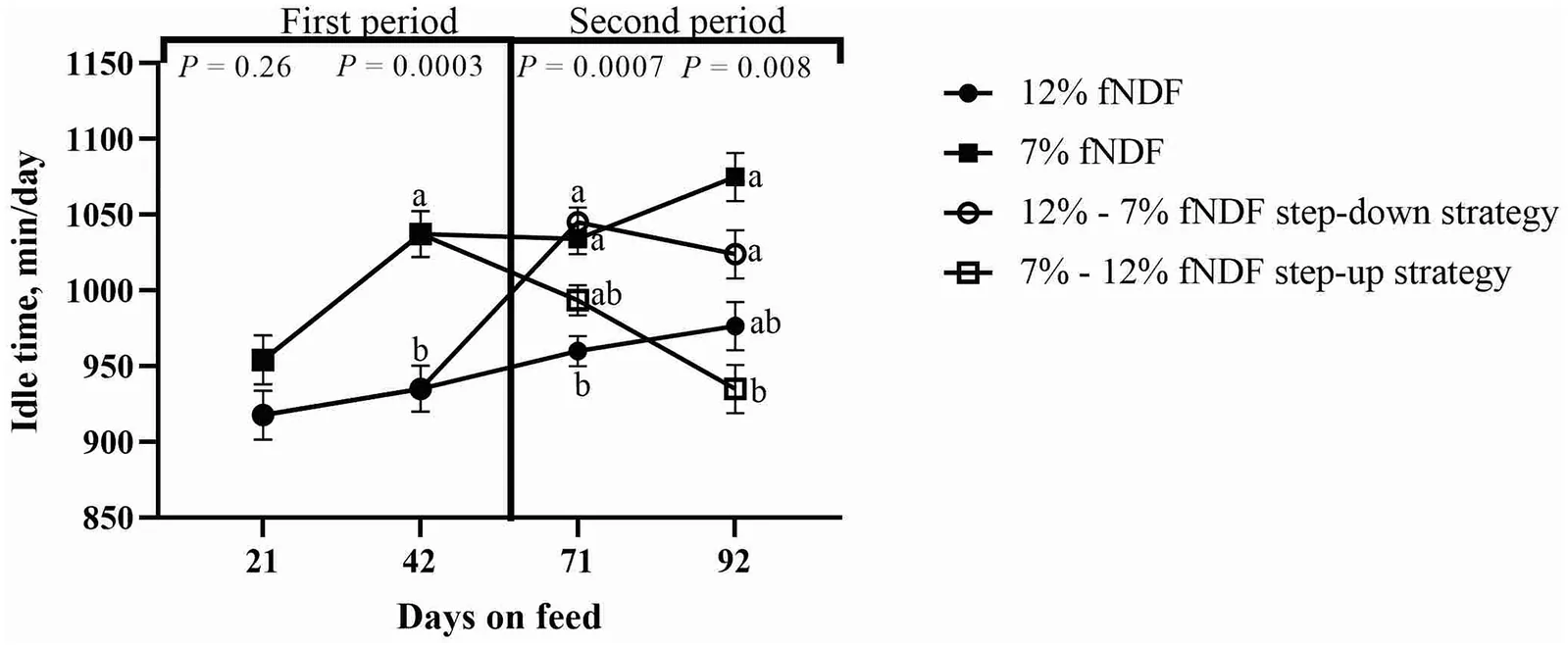
Minutes of idle time per day of Nellore bulls fed diets containing 12% or 7% forage neutral detergent fiber (fNDF), either continuously or with dietary transitions (12%–7% fNDF step-down strategy or 7%–12% fNDF step-up strategy), measured at 21, 42, 71, and 92 days of feedlot. 12% fNDF—constant 12% fNDF throughout the 100 days. 7% fNDF—constant 7% fNDF throughout the 100 days. 12%–7% fNDF step-down strategy—step-down strategy fNDF, from 12% in period 1 to 7% in period 2; 7%–12% fNDF step-up strategy—step-up strategy fNDF, from 7% in period 1 to 12% in period 2. In the first period (0–50 days), animals received diets containing 12% fNDF (two pens) or 7% fNDF (two pens), and results were averaged across pens with the same fNDF level. ^a,b^Means within a row with different superscript letters differ (*P* < 0.05) according to Tukey’s test.

Regarding daily idle time (Fig. 5), bulls fed the 12% fNDF diet exhibited shorter idle times at 42 and 71 d (*P* < 0.01) compared to those fed 7% fNDF. In the second period, at 92 d, animals assigned to the 7%–12% Step-up strategy exhibited the shortest idle time (*P* < 0.01), whereas those on the 12%–7% Step-down and continuous 7% fNDF strategies exhibited the longest idle times (*P* < 0.01).

**Figure 6:**
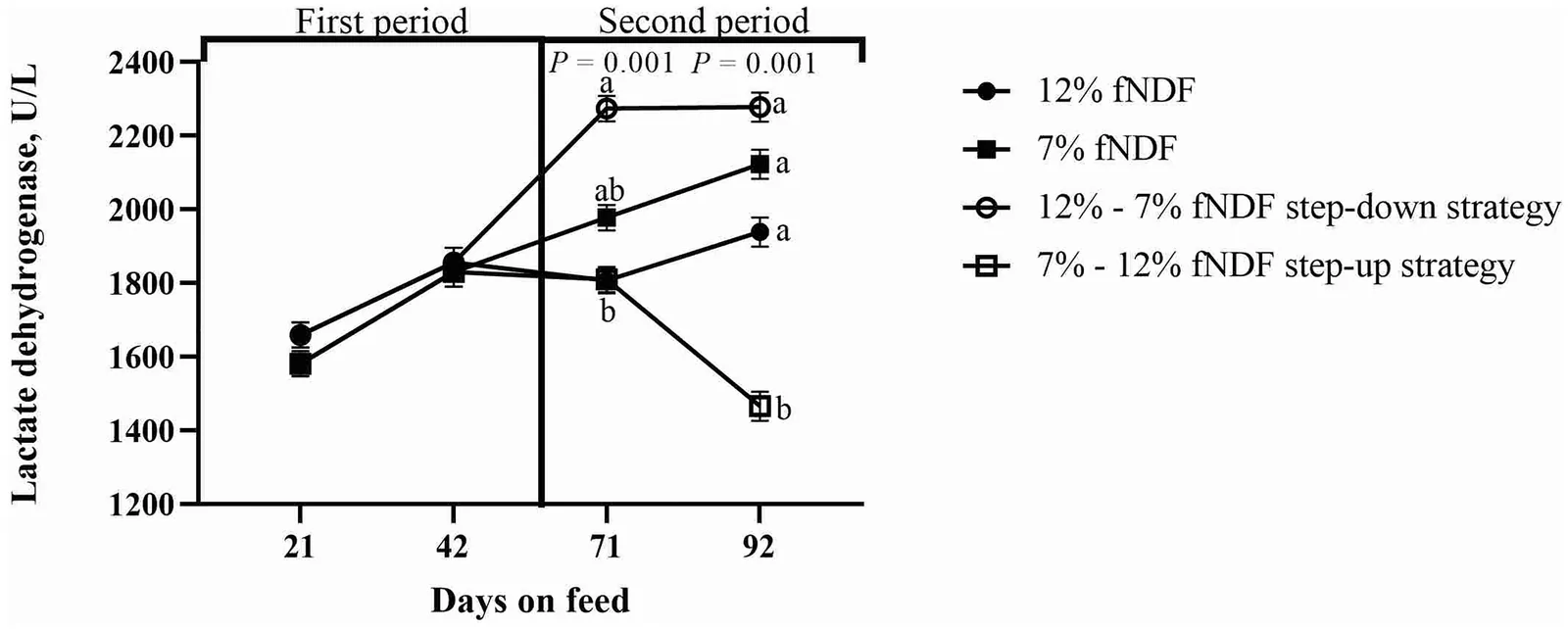
Concentration of lactate dehydrogenase in the blood of Nellore bulls fed diets containing 12% or 7% forage neutral detergent fiber (fNDF), either continuously or with dietary transitions (12%–7% fNDF step-down strategy or 7%–12% fNDF step-up strategy), measured at 21, 42, 71, and 92 days of feedlot. 12% fNDF—constant 12% fNDF throughout the 100 days. 7% fNDF—constant 7% fNDF throughout the 100 days. 12%–7% fNDF step-down strategy—step-down strategy fNDF, from 12% in period 1 to 7% in period 2; 7%–12% fNDF step-up strategy—step-up strategy fNDF, from 7% in period 1 to 12% in period 2. In the first period (0–50 days), animals received diets containing 12% fNDF (two pens) or 7% fNDF (two pens), and results were averaged across pens with the same fNDF level. ^a,b^Means within a row with different superscript letters differ (*P* < 0.05) according to Tukey’s test.

Serum LDH concentrations did not differ (*P* > 0.05) during the first period (Fig. 6). However, at 71 d, bulls fed the continuous 12% fNDF and the 7%–12% Step-up exhibited lower blood LDH concentrations (*P* < 0.01) compared to those on the 12%–7% Step-down strategy. At 92 d, bulls subjected to the 7%–12% Step-up transition maintained a significant reduction in serum LDH concentration (*P* < 0.01) compared to other treatments. Haptoglobin and serum amyloid A concentrations (Table 5) did not differ among treatments throughout the feedlot period (*P* > 0.05).

**Table 5:** Concentration of haptoglobin and serum amyloid-A of nellore bulls fed different fNDF strategies in the feedlot.

Item	Treatment^a^				SEM^b^	*P*-value
	12% fNDF	7% fNDF	12%–7% fNDF step-down strategy	7%–12% fNDF step-up strategy		
**Haptoglobin (mg/mL)**						
** 21 d**	0.443	0.485	–	–	0.02	0.45
** 92 d**	0.210	0.284	0.427	0.403	0.03	0.08
**Amyloid A (mg/L)**						
** 21 d**	41.0	51.9	–	–	2.78	0.18
** 92 d**	45.05	53.58	51.37	59.58	2.28	0.38

a 12% fNDF^1^—constant 12% fNDF throughout the 100 days; 7% fNDF^2^—constant 7% fNDF throughout the 100 days; 12%–7% fNDF step-down strategy³—step-down strategy fNDF, from 12% in period 1 to 7% in period 2; 7%–12% fNDF step-up strategy^4^—step-up strategy fNDF, from 7% in period 1 to 12% in period 2; In the first period (0–50 days), animals received diets containing 12% fNDF (two pens) or 7% fNDF (two pens), and results were averaged across pens with the same fNDF level.

b SEM^5^, standard error of the mean.

**Figure 7:**
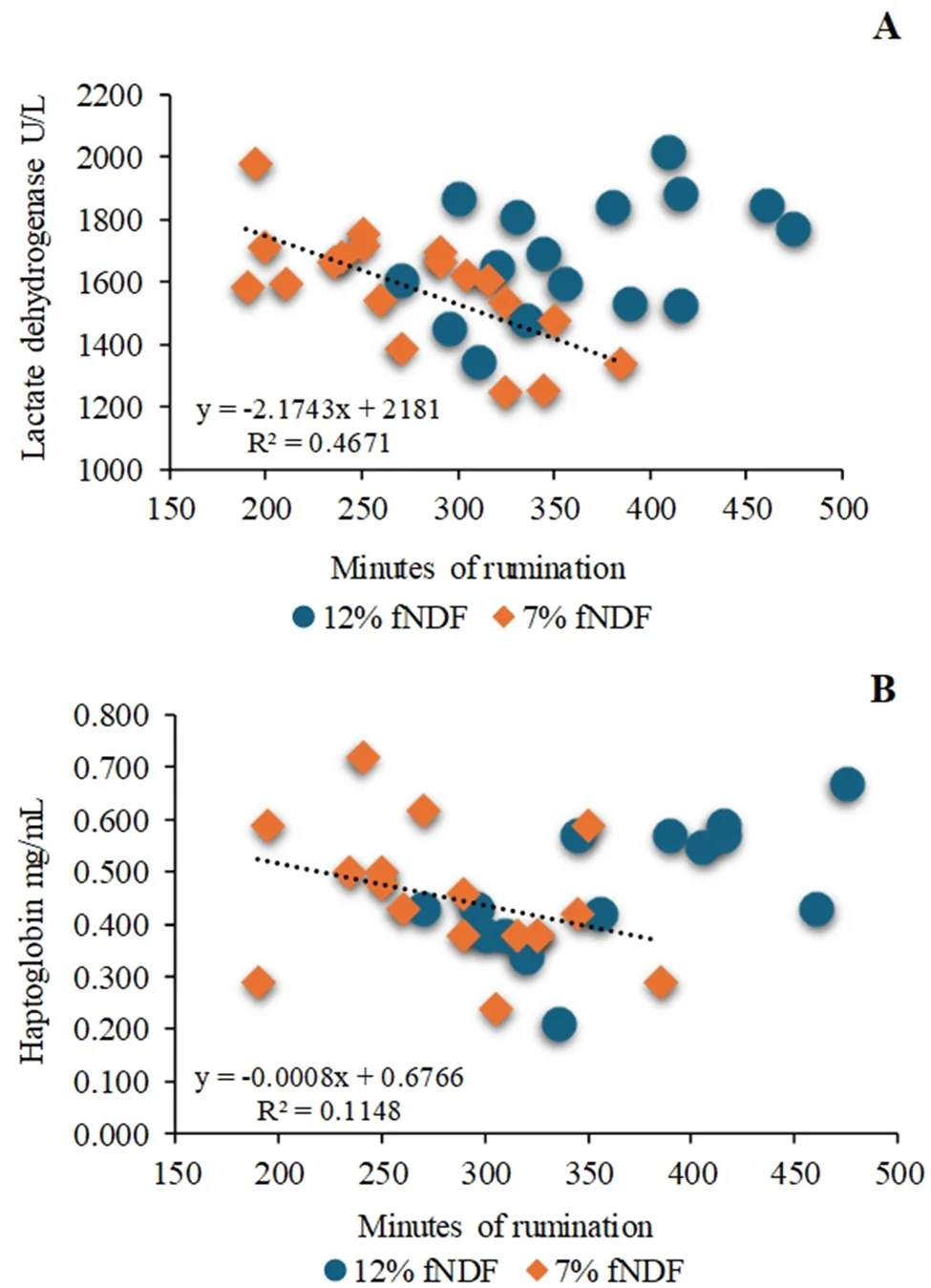
Relationship between rumination time (min/day) and A) lactate dehydrogenase (LDH) and B) haptoglobin concentrations in Nellore bulls fed diets containing 7% or 12% forage neutral detergent fiber (fNDF) during the feedlot.

The relationship between rumination time and the inflammatory markers LDH (A) and haptoglobin (B) during the first period is presented in Fig. 7. The regression model was significant (*P* < 0.01), showing a significant crossover interaction between rumination time and dietary fNDF level (*P* < 0.01) for LDH. The model is described by the equation:


LDH=1272.17+908.86×(7% fNDF)+1.1427×rumi-3.3170×(7% fNDF×rumi).

The intercept (1272.17) represents the expected LDH concentration at zero rumination time for animals fed 12% fNDF. Bulls fed the 7% fNDF diet had greater LDH concentrations at zero rumination time (+908.86 units) compared to those fed 12% fNDF. In the 12% fNDF treatment, LDH increased with rumination time (β = 1.14; *P* = 0.0417). In contrast, for bulls receiving the 7% fNDF diet, the slope was negative (β = −2.17), indicating that LDH concentration decreased linearly as rumination time increased (*P* < 0.01).

Similarly, a significant interaction between rumination time and dietary fNDF level was observed for haptoglobin (*P* < 0.01). The model is described by the equation:


Haptoglobin=0.02476+0.6519×(7% fNDF)+0.001217×rumi-0.00202×(7% fNDF×rumi).

The intercept (0.02476) represents the expected haptoglobin concentration at zero rumination time for animals fed 12% fNDF. Animals receiving the 7% fNDF diet had higher baseline haptoglobin concentrations (+0.6519 units). In the 12% fNDF treatment, haptoglobin concentration increased slightly with rumination time (β = .001217; *P* = 0.0212). In contrast, in animals fed 7% fNDF, the slope was negative (β = −0.000803), demonstrating that haptoglobin concentration decreased linearly with increasing rumination time (*P* = 0.02).

## Discussion

The results of this study demonstrate that variations in dietary fNDF levels for Nellore bulls affect rumination time, chewing, idleness, and inflammatory responses throughout the feedlot. The difference in rumination time between the 12% and 7% fNDF treatments throughout the feedlot suggests that greater fNDF inclusion may promote greater masticatory and salivary stimulation, potentially improving ruminal buffering capacity (Beauchemin 2018), although ruminal pH was not directly measured in this study.

Previous studies have demonstrated that increasing the concentration of physically effective fiber stimulates chewing and rumination, enhances salivary buffering, and contributes to maintaining a higher ruminal pH, thereby reducing the risk of subacute ruminal acidosis (Mertens 1997; Owens et al. 1998; Beauchemin 2018). However, most of these studies evaluated fixed dietary fiber concentrations, whereas information regarding sequential changes in dietary fNDF during the feedlot period remains scarce, particularly in Nellore cattle. Although ruminal pH was not measured in the present study, the greater rumination observed in bulls receiving higher dietary fNDF is consistent with the established physiological mechanism linking chewing activity to improved ruminal buffering capacity.

Bulls subjected to a decrease in fNDF from 12% to 7% exhibited reduced eating, chewing, and rumination times, whereas increasing fNDF from 7% to 12% increased rumination time and reduced idle time. These findings are consistent with previous studies demonstrating that chewing activity increases with dietary NDF concentration because structural carbohydrates require greater mastication during both feed consumption and rumination (Grant and Cotanch 2023). Therefore, changes in the fiber proportion in cattle diets influence animal behavior.

Low dietary NDF inclusion in feedlot diets should be approached with caution, as it may reduce the ruminal mat, decrease rumination stimulation, shorten chewing duration, and allow ruminal pH to drop toward the subacute acidosis threshold of approximately 5.8 (Mertens 1997; Plaizier et al. 2008). Greater energy concentrations are more cost-effective than forage feeds; however, diets with a greater concentration of ruminally fermentable carbohydrates pose challenges to ruminal health (Gozho et al. 2005).

Lactate dehydrogenase concentration is one of the blood parameters evaluated, along with other acute-phase proteins, as an indicator of animal welfare and of metabolic disorders. When cellular contents of the tissue are released into the plasma, as commonly occurs in cases of cellular injury, the serum LDH isoenzyme pattern changes, reflecting the profile of the affected organ or tissue. This parameter has diagnostic relevance (Klein et al. 2020).

After switching diets, animals receiving 12% fNDF exhibited lower serum LDH concentrations than those fed lower-fiber diets. Because LDH is widely distributed among tissues, total circulating LDH activity should be interpreted as a nonspecific indicator of altered cellular integrity or tissue turnover rather than as direct evidence of metabolic stress, liver injury, or acidosis (Klein et al. 2020). Therefore, the lower LDH concentrations observed in bulls fed 12% fNDF may reflect differences in physiological responses associated with dietary fiber level. However, additional measurements, such as tissue-specific biomarkers and ruminal pH, would be necessary to elucidate the underlying mechanisms.

Bevans et al. (2005) evaluated two adaptation protocols, rapid and gradual, and found differences in plasma LDH concentration in beef cattle on the fourth day of the protocols. Cattle fed the submitted diet and subjected to a faster adaptation protocol had an average LDH concentration of 4,524 U/L, while those subjected to a slower adaptation protocol had an average of 3,750 U/L, indicating the presence of subacute acidosis (SARA) in animals offered diets rich in concentrate.

In the present study, the LDH level increased throughout the feedlot and remained stable among animals receiving 12% fNDF. This finding suggests a persistent influence of fNDF content on LDH concentration regulation throughout the feedlot, reinforcing the importance of these data for understanding the metabolic effects associated with different dietary formulations. Although SAA and haptoglobin concentrations did not differ significantly among treatments, the numerically greater values observed in bulls fed 7% fNDF are consistent with reports of increased acute-phase protein concentrations during SARA (Gozho et al. 2005; Emmanuel et al. 2008; Khafipour et al. 2009).

Pereira et al. (2023) reported that feedlot Simmental cattle fed fiber-free diets observed elevated serum concentrations of amyloid A and haptoglobin compared to animals fed a diet containing 10% wheat straw, either chopped or pelleted. This emphasizes that when animals are exposed to greater energy diets, the ruminal environment becomes more prone to acidosis, which is correlated with increased levels of acute phase proteins such as serum amyloid A and haptoglobin (Plaizier et al. 2008). Rumination time is highly correlated with animal welfare. In the first 50 days of feedlot, the longer time of rumination was associated with lower concentrations of LDH and haptoglobin.

Rumination time is also associated with greater weight gain. Marchesini et al. (2018) used rumination minutes as early indicators of health and performance in beef cattle during the beginning of the feedlot. Sensor collars were used to monitor activity (bits) and rumination (minutes) in 214 Charolais steers during the first 70 days after arrival at the feedlot units. The bulls fed 7% fNDF exhibited smaller weight gain, smaller daily rumination, and greater variation in daily rumination during the first feedlot period. Thus, unstable rumination and lower activity variability are associated with poorer productive performance (Souza et al. 2026).

This study reinforces the importance of fNDF in feedlot cattle diets, demonstrating that changes in the fNDF directly impact on the animals’ ingestive behavior and metabolism. The findings indicate that reduced fiber intake was associated with physiological alterations, including changes in selected blood biomarkers, suggesting potential metabolic challenges associated with reduced dietary fiber. Therefore, diet formulation should strike a balance between fiber and concentrate levels to maximize production efficiency without compromising animal welfare.

A limitation of the present study is that ruminal pH and other ruminal fermentation parameters were not measured. Therefore, although changes in ingestive behavior and blood biomarkers may suggest differences in physiological responses associated with dietary fiber level, direct inferences regarding ruminal acidosis or ruminal environment should be interpreted with caution. Future studies including direct ruminal measurements are needed to confirm the mechanisms underlying the responses observed in the present study.

## Conclusion

Dietary forage NDF levels influenced ingestive behavior and selected blood biomarkers in Nellore bulls during the feedlot period. Diets containing 12% fNDF consistently increased rumination and chewing activity while reducing idle time, indicating a behavioral pattern consistent with greater rumination activity. In addition, lower lactate dehydrogenase concentrations observed under 12% fNDF indicate differences in physiological responses associated with dietary fiber level.

## Abbreviations

ADG: Average daily gain
BW: Body weight
d: Day(s)
DM: Dry matter
DMI: Dry matter intake
fNDF: Forage neutral detergent fiber
12% fNDF: constant 12% fNDF throughout the 100 days
7% fNDF: constant 7% fNDF throughout the 100 days
12% -: 7% fNDF step-down strategy—12% in period 1 to 7% in period 2
7% -: 12% fNDF step-up strategy—7% in period 1 to 12% in period 2
GLM: General linear model
h: Hour(s)
IBW: Initial body weight
LDH: Lactate dehydrogenase
min: Minute(s)
NDF: Neutral detergent fiber
SAA: Serum amyloid A
SEM: Standard error of the mean
